# Bicarbonate-Triggered In Vitro Capacitation of Boar Spermatozoa Conveys an Increased Relative Abundance of the Canonical Transient Receptor Potential Cation (TRPC) Channels 3, 4, 6 and 7 and of CatSper-γ Subunit mRNA Transcripts

**DOI:** 10.3390/ani12081012

**Published:** 2022-04-13

**Authors:** Estíbaliz Lacalle, César Consuegra, Cristina A. Martínez, Manuel Hidalgo, Jesús Dorado, Felipe Martínez-Pastor, Manuel Álvarez-Rodríguez, Heriberto Rodríguez-Martínez

**Affiliations:** 1 Department of Biomedical & Clinical Sciences (BKV), BKH/Obstetrics & Gynecology, Faculty of Medicine and Health Sciences, Linköping University, SE-581-85 Linköping, Sweden; estibaliz.1302@gmail.com (E.L.); cconsuegra93@hotmail.com (C.C.); cristina.martinez-serrano@liu.se (C.A.M.); heriberto.rodriguez-martinez@liu.se (H.R.-M.); 2 Institute of Animal Health and Cattle Development (INDEGSAL), Universidad de León, 24071 León, Spain; felipe.martinez@unileon.es; 3 Veterinary Reproduction Group, Department of Medicine and Animal Surgery, Faculty of Veterinary Medicine, University of Cordoba, 14071 Cordoba, Spain; mhidalgo@uco.es (M.H.); jdorado@uco.es (J.D.); 4 Department of Molecular Biology (Cell Biology), Universidad de León, 24071 León, Spain; 5 Department of Animal Health and Anatomy, Universitat Autònoma de Barcelona, 08193 Bellaterra, Spain

**Keywords:** calcium channels, catSper subunits, TRPC, pig, capacitation

## Abstract

**Simple Summary:**

The detection of sub-fertile boars has been a difficult task, and despite their prevalence being low, its impact is very significant because it implies economic drawbacks for artificial insemination (AI) centers and farms. Unfortunately, some crucial reproductive processes fall beyond the routine analysis performed in the porcine model, such as sperm capacitation, which is a necessary event for fertilization. A synergistic action of bicarbonate (HCO_3_^−^) with calcium (Ca^2+^) is needed to achieve capacitation. The transport of Ca^2+^ is mediated by CatSper channels and Canonical Transient Potential Channels (TRPC). We quantified mRNA transcripts of different subunits of CatSper (β, γ and δ) and TRPC (1, 3, 4, 6 and 7) before and after in vitro capacitation by HCO_3_^−^ ions. Our results showed that in vitro capacitation using HCO_3_^−^ increases the relative abundance of mRNA transcripts of almost all subunits of Ca^2+^ channels, except *CatSper*-δ and *TRPC1*, which were significantly reduced. More studies are needed to elucidate the specific roles of the TRPC channels at a physiological and functional level.

**Abstract:**

Sperm capacitation is a stepwise complex biochemical process towards fertilization. It includes a crucial early calcium (Ca^2+^) transport mediated by CatSper channels and Canonical Transient Potential Channels (TRPC). We studied the relative abundance of mRNA transcripts changes of the *CatSper* β, γ and δ subunits and *TRPC*-channels 1, 3, 4, 6 and 7 in pig spermatozoa, after triggering in vitro capacitation by bicarbonate ions at levels present in vivo at the fertilization site. For this purpose, we analyzedfive5 ejaculate pools (from three fertile adult boars) before (control-fresh samples) and after in vitro exposure to capacitation conditions (37 mM NaHCO_3_, 2.25 mM CaCl_2_, 2 mM caffeine, 0.5% bovine serum albumin and 310 mM lactose) at 38 °C, 5% CO_2_ for 30 min. In vitro capacitation using bicarbonate elicits an increase in the relative abundance of mRNA transcripts of almost all studied Ca^2+^ channels, except *CatSper*-δ and *TRPC1* (significantly reduced). These findings open new avenues of research to identify the specific role of each channel in boar sperm capacitation and elucidate the physiological meaning of the changes on sperm mRNA cargo.

## 1. Introduction

Spermatozoa undergo different maturation steps while they interact with different environments throughout their life [1]. Although they have come to be mature and motile in the epididymis, these male gametes unable to fertilize oocytes upon ejaculation because they must reside in the reproductive female tract for a period of time and interact with its environment to be able to fertilize [2].

Sperm capacitation is a gradual and necessary event for fertilization, which occurs during the journey of the spermatozoa through the female reproductive tract in vivo [3,4]. This process is characterized by controlled biochemical changes, including destabilization of the plasma membrane [3]. These changes should ultimately prepare spermatozoa to reach and bind to the zona pellucida (ZP) of the oocyte, undergo acrosome reaction (AR), and eventually penetrate the oocyte [5]. The capacitation process in the porcine model occurs mainly in the ampulla, where increasing extracellular bicarbonate enters spermatozoa by the concentration gradient, resulting in initial destabilizing membrane modifications [4]. The synergistic action of HCO_3_^−^ with calcium (Ca^2+^) is needed to achieve capacitation since HCO_3_^−^ stimulates Ca^2+^ uptake in porcine spermatozoa [6].

Sperm capacitation occurs through two complementary pathways [7]. One of them is dependent on HCO_3_^−^, which elevates cAMP levels. cAMP increase induces PKA, producing tyrosine phosphorylation in the proteins (cAMP/PKA pathway). Boar spermatozoa require HCO_3_^−^ to capacitate [3], an ion that exists in a low concentration in the epididymis (3–4 mM), helping to maintain sperm quiescence [8]. Levels increase 9–10-fold in the seminal plasma (up to 20–30 mM in the sperm-rich fraction), which is known to stimulate but not capacitate spermatozoa (in physiological conditions) [8,9,10]. This concentration also varies among fractions, being 14–17 mM in the pre-sperm fraction and sperm-rich fraction while increasing to >30 mM in the post sperm-rich fraction [10]. However, in the oviduct, the HCO_3_^−^ concentration increases from the proximal to the distal portion, which is the upper segment (where spermatozoa are closer to the fertilization place). Whereas bicarbonate is still low in the isthmus, it considerably increases to the ampulla (10 to 33 mM) according to in vivo measurements. In the ampulla, bicarbonate enters spermatozoa by the concentration gradient, resulting in changes in sperm plasma membrane [4], acting synergically with Ca^2+^ and inducing capacitation. HCO_3_^−^ stimulates Ca^2+^ uptake in porcine sperm [6], and together, they activate adenylate cyclase, increasing intracellular cyclic adenosine monophosphate [cAMP]i. The increase in [cAMP]i activates cAMP-dependent protein kinases (PKA), resulting in the phosphorylation of key effectors for capacitation [9,10,11,12].

Alternatively, spermatozoa can achieve capacitation by a c-Src-dependent pathway. c-Src, a member of the Src family kinase (SFK), decreases the activity of serin/threonine phosphatases and increasing the levels of tyrosine phosphorylation (SFK/phosphatase pathway) [7]. The capacitation events increase protein–tyrosine phosphorylation in the sperm tail [12,13], which appears to be related to the accompanying hyper-activation of sperm motility [13,14,15]. There is also an activation of protein kinases in the sperm head, increasing membrane fluidity and lipid reorganization [16,17]. These alterations in the sperm head allow cholesterol removal through serum albumin, a cholesterol acceptor that acts only on the subpopulation of spermatozoa sensitive to bicarbonate [16]. Albumin extracts cholesterol from the plasma membrane, increasing the fluidity of the sperm membrane and ion permeability (Na^+^ entry and K^+^ output). Then, there is a sperm membrane hyperpolarization and an increase in Ca^2+^ levels inside spermatozoa, resulting in increased intracellular pH and protein–tyrosine phosphorylation [18]. In addition, there is a redistribution of tyrosine-phosphorylated proteins localized in the acrosomal region [16].

Presently, knowing the composition of the female tract place where sperm capacitation occurs and the molecules involved in either of the two pathways of the capacitation process, in vitro capacitation can be performed in most of the mammalian species. It is accepted that a capacitation medium should contain ions, including HCO_3_^−^ and Ca^2+^ and bovine serum albumin (BSA) [19,20].

This chain of events occurring through the capacitation pathways induces membrane hyperpolarization, which later activates diverse calcium channels [21,22,23]. The regulation of Ca^2+^ influx is governed through sperm-specific Ca^2+^ channels (CatSper) and other ion channels such as canonical transient receptor potential channels (TRPC) [24].

The CatSper channel family is the most studied Ca^2+^ channel in spermatozoa. CatSper is a sperm-specific Ca^2+^ permeable channel located in the membrane of the flagellum principal piece, which helps the influx of this cation during capacitation [25] through changes in membrane potential and pH [26,27]. It has ten subunits with four pore-forming α subunits (CatSper 1–4) and, at least, six auxiliary subunits: β, γ, δ, Ɛ, ζ and EFCAB9 [28], although other previous studies describe three [29] (β, γ and δ) or five (β, γ, δ, Ɛ and ζ) accessory proteins [30]. The CatSper channel complex is organized in quadrilateral longitudinal nanodomains, running down the principal piece of the flagellum [24]. These channels can trigger signal transduction factors that are generally required for initiating the cAMP-PKA signaling pathway and subsequent steps in sperm capacitation [31]. CatSper mutations in humans have been associated to infertility [32,33], and it is established that they are essential for egg coat penetration and fertility in mammals [33], showing their importance for sperm function. In addition to mammals, these channels are also present in reptiles, tunicates, echinoderms and cnidarians [34].

Canonical Transient Receptors Potential channels (TRPC) also affect ion influx, such as Ca^2+^ (but also including K^+^ and Na^+^) [35]. TRPC can be activated by voltage, although they are not voltage-gated channels [36]. Up to seven *TRPC* (*TRPC1-7*) genes have been identified in mammals, being related with the phospholipase C (PLC)-dependent Ca^2+^ influx [37]. These seven TRPC subunits are functionally localized in spermatozoa [35], although their distribution varies between different species of mammals. TRPC3, one of the most studied TRPC subunits, appears in the acrosome of human, mouse and goat sperm but only in the midpiece of human and goat sperm. However, in mouse sperm, TRPC3 protein expression appears also in the distal segment of the flagellum [35,38,39].

The complete mapping of the ejaculate parameters could define fertility when retrospectively related to the observed sire fertility [40], including the omics of both spermatozoa [40,41,42] and seminal plasma (SP) [43]. This combination of analyses should help determine suitable non-invasive molecular biomarkers to identify different fertility potentials in the pig using SP [43] or RNA present in spermatozoa [44]. The study of the proteins, genes and metabolites involved, as well as the impact of epigenetic modifications as biomarkers, may be used to diagnose infertility also in human andrology [45].

mRNA transcripts in spermatozoa have been proposed as a possible marker for male fertility [44,46]. Given the importance of Ca^2+^ channels for sperm functionality and fertility, our objective was to determine possible changes in the relative abundance of mRNA transcripts of sperm Ca^2+^ channels in pig spermatozoa, after in vitro capacitation was triggered by bicarbonate at levels present in vivo at the site of fertilization. Due to the relevance of this channels on calcium signaling, we studied the CatSper subunits (*CatSper* β, γ and δ subunits) and various TRPC channels (*TRPC* 1, 3, 4, 6 and 7) using commercially available primers for pig. We assessed other sperm variables such as motility, membrane fluidity and PKA and cAMP pathways to find a possible link with mRNA transcript abundance.

## 2. Materials and Methods

All chemicals and reagents were purchased from Sigma-Aldrich Co. (St. Louis, MO, USA) unless otherwise stated.

### 2.1. Ethics Statement

Animal husbandry and all experimental and analytical procedures were performed in compliance with European Community and Swedish legislation (Directive 2010/63/EU; Swedish SJVFS 2017:40), and approvals by the “Regional Committee for Ethical Approval of Animal Experiments” were obtained (Linköpings Djurförsöksetiska nämnd), Linköping, Sweden (Dnr 75-12; ID1400; 03416-2020).

### 2.2. Sperm Handling and In Vitro Capacitation

Boar ejaculates were supplied as commercial AI doses (5 batches of 3 same boars per pool) from Svenska Köttföretagen AB, Hållsta, Sweden extended in Hampshire Longlife to 4.8 × 10^9^ total spermatozoa/dose, each batch containing a pool of three different breeding boars of proven fertility and semen quality (>80% motility, <15% total sperm abnormalities), and stored at 17 °C, as recommended for AI purposes. All incoming sperm samples were re-examined for motility (QualiSperm, Biophos AG, Pfäffikon, Switzerland) [47] and sperm membrane stability using YO-PRO-1 labeling. All samples yielding > 80% motility and viability were accepted.

Spermatozoa that were used for in vitro capacitation group were washed twice in phosphate-buffered saline (PBS, 300× *g*, 5 min) and incubated in a capacitation medium (37 mM NaHCO_3_, 2.25 mM CaCl_2_, 2 mM caffeine, 0.5% bovine serum albumin and 310 mM lactose) at 38 °C, 5% CO_2_, for 30 min [48]. Following centrifugation (5000× *g*, 5 min), the sperm pellets of both control and in vitro capacitated samples were stored at -80 °C until further analysis (Ultra Low Freezer; Haier Inc., Qingdao, China) for analyzing all parameters: sperm evaluation (motility, viability and mitochondrial status) mRNA expression, cAMP and PKA.

### 2.3. Experimental Design

Both capacitated and non-capacitated sperm samples were evaluated by measuring several sperm quality parameters: Motility, velocity and membrane fluidity assessment were evaluated in non-capacitated samples and just after the samples were capacitated. After that, pellets were frozen at −80 °C. The other parameters, including PKA and cAMP levels and the relative expression of the different subunits from Ca^2+^ channels, were evaluated from these stored pellets at −80 °C.

### 2.4. Sperm Evaluation

A 5 µL drop of each sperm sample was placed in a pre-warmed (38 °C) slide, covered with an 18 × 18 mm coverslip and examined under phase-contrast microscopy by using a computerized system (Qualisperm^TM^ software, AKYmed, Cheseaux-sur-Lausanne, Switzerland) [47], which was connected via a CMOS camera (UEye, IDS Imaging Development Systems GmbH, Ubersulm, Germany) to an upright Zeiss Axio Scope A1 light microscope using a 10X phase contrast objective (Carl Zeiss, Stockholm, Sweden). An algorithm calculates the number of fluctuations in each pixel by correlation function, which is used to calculate the speed (velocity) distribution. This system runs in high throughput mode (usually four fields per minute), analyzing > 2000 spermatozoa/field. The following sperm motility parameters were obtained from the speed (µm/s): total motility (%), fast progressive (%), slow progressive (%), non-progressive (%) and immotile (%) spermatozoa.

### 2.5. Capacitation Status

Capacitation status was assessed using the fluorochrome M540 in a fluorescence microscope and the analysis of PKA and cAMP by specific kits.

#### 2.5.1. Membrane Fluidity Assessment

A combination of two fluorescent probes in PBS was used to evaluate cell physiology: YO-PRO-1 (75 nM) for viability and Merocyanine 540 (M540, 2 µM) for capacitation status. Merocyanine 540 (M540) is an impermeant fluorescent probe that preferentially binds to membranes with lower cholesterol: phospholipids ratio, i.e., under destabilization, and YO-PRO-1 stains the nuclei of apoptotic or dead sperm with increased plasma membrane permeability. After 15 min at 37 °C in the dark, 200 spermatozoa of each single boar from each treatment (control and capacitated) were counted using a fluorescence microscope at 40× magnification (Eclipse Ti; Nikon, Stockholm, Sweden). Three fluorescence patterns were identified: Spermatozoa showing green fluorescence were considered apoptotic or dead; those showing yellow fluorescence were considered viable and capacitated; spermatozoa without fluorescence were considered viable non-capacitated. We recorded the viable sperm (YO-PRO-1^−^) and viable and capacitated (live-capacitated) sperm ratio (bright M540 fluorescence and negative YO-PRO-1 fluorescence/total sperm count × 100).

#### 2.5.2. cAMP and PKA Assessment

Each capacitated/non capacitated semen pellet (100 × 10^6^ spermatozoa) was resuspended in 1 mL of PBS. Then, each sample was split into two 1.5 mL tubes with 500 µL each (50 × 10^6^ spermatozoa). One of them was directed to cAMP analysis by Direct Immunoassay, and the other one was directed to PKA analysis by Kinase Activity assessment.

For cAMP evaluation, the cAMP direct immunoassay fluorometric kit (abcam, Cambridge, United Kingdom; ab138880) was used, following the manufacturer’s specifications. In brief, all samples (50 × 10^6^ spermatozoa), control and in vitro capacitated samples were centrifuged at 3000× *g* for 5 min at room temperature (RT); then, the supernatant was discarded, and the pellet was resuspended by adding 100 µL of Cell Lysis Buffer in each well of the plate and incubated at RT for 10 min. Samples were centrifuged for 5 min at top speed, and the supernatant was collected and transferred to a new tube that was kept on ice. A volume of 75 µL of standard and sample was added to the wells of the anti-cAMP coated 96-well plate and incubated at RT for 5–10 min. After this time, 25 µL/well of 1X HRP-cAMP conjugate was added to each standard and sample well. Then, the plate was incubated at RT for 2 h on a plate shaker and washed 4 times with 200 µL Wash Solution. Finally, 100 µL AbRed Working Solution was added into each standard and sample well, incubating the plate at RT for 1 h protected from light. Fluorescence change was measured in a microplate reader set to top read mode at Ex/Em = 540/590 nm (cutoff 570 nm).

For PKA evaluation, we used the PKA Kinase Activity Assay Kit (abcam, Cambridge, United Kingdom; ab139435) according to the manufacturer’s instructions. In brief, all samples (50 × 10^6^ spermatozoa), control and in vitro capacitated sperm were centrifuged at 3000× *g* for 5 min at RT, and the pellet was resuspended in 100 µL of RIPA. After sonication of the samples (10 s twice) and 5 s vortexing, the samples were kept on ice for 30 min. Then, the lysate was centrifuged 13,000× *g* for 15 min at 4 °C. After recovery of a clear supernatant, the samples were kept on ice. Wells were previously prepared with 50 µL Kinase Assay Dilution Buffer at RT for 10 min. Then, 30 µL Kinase dilution buffer was added to the blank wells, samples (30 µL) and controls (30 µL) to the appropriate wells. An amount of 10 µL of diluted ATP to each well (except blank) initiates the reaction, after incubation at 30 °C for 90 min. Then, 40 µL of phospho-specific antibody was added to each well, except blank, and the plate was incubated at RT for 60 min. All wells were washed 4 times with 100 µL 1X Wash Buffer. Afterward, 40 µL of diluted anti-rabbit IgG-HRP conjugated was added to each well except blank, and the plate was incubated at RT for 30 min and washed 4 times with wash buffer. Thereafter, 60 µL TMB solution was added and incubated for 45 min at RT. Finally, 20 µL stop solution was added to each well to stop the reaction, and the optical density (OD) at 450 nm was measured.

### 2.6. RNA Extraction and qPCR Analyses of Calcium Channels

Total RNA was extracted from the sperm samples using an RNeasy micro kit (Qiagen, Venlo, The Netherlands) following the manufacturer’s instructions. The RNA concentration of the extracts was determined from the absorbance of 260 nm with Thermo Scientific NanoDropTM 2000 (Fisher Scientific, Gothenburg, Sweden). The High-Capacity RNA-to-cDNA™ Kit (Fisher Scientific, Gothenburg, Sweden) was used to synthesize the first-strand cDNA for quantitative polymerase chain reaction (qPCR) analyses (CFX96™; Bio-Rad Laboratories, Inc; Hercules, CA, USA). The gene relative abundance levels were quantified using the Pfaffl method, ΔΔCt method (Pfaffl, 2001). The *GAPDH* gene and commercial gene-specific PCR primers for porcine samples were used (glyceraldehyde-3-phosphate dehydrogenase) (PrimePCR™SYBR^®^ Green Assay: *GAPDH*, Pig; Bio-Rad Laboratories, Inc; Hercules, CA, USA). We included NTC controls in all plates, obtaining in all cases an indetectable presence of the products after qPCR analysis (>40 cycles).

PowerUp SYBR Green Master Mix (2×) (Applied Biosystems, Foster City, CA, USA) was used for PCR reactions. The final reaction volume was 10 μL (2 μL of cDNA (25 ng), 5 μL of 2X Master mix, 1 μL of each primer (500 nM) and 1 μL of dH2O). The following PCR conditions were used: initial UDG activation of 50 °C for 2 min and a previous denaturation of 95 °C for 2 min. Those steps were followed by 40 cycles of 5 s of denaturation at 95 °C and 30 s of extension and annealing at 60 °C. qPCRs were run in duplicate for each gene per sample. All primers (TRPC1, TRPC3, TRPC4, TRPC6, TRPC7, CatSper-β, CatSper-δ and CatSper-γ) were commercial from BIORAD (PrimePCR™SYBR^®^ Green Assay, Pig; Bio-Rad Laboratories, Inc; Hercules, CA, USA). Target relative gene abundance was normalized with the reference gene *GAPDH*. By examining the results of the number of cycles in qPCR, we verified that there are no significant differences in the *GAPDH* cycles before and after sperm in vitro capacitation. In addition, the number of cycles increased in all TRPCs and CatSper subunits, although without significant differences. Moreover, additional validation was the same concentration of total sperm cells in both measurement time points before and after capacitation.

### 2.7. Statistical Analysis

Normal distribution and homoscedasticity of the data were analyzed using the Shapiro–Wilk normality test and Levene’s test. Non-normal data distribution was reached using arcsin(x) transformation prior to analysis. R version 3.6.1 [49] was used to conduct statistical analyses, with nlme [50] to perform linear mixed effects (LME) models and multcomp [51] to perform pairwise comparisons adjusted by Tukey’s test. The threshold for significance was set at *p* < 0.05. Data are presented as mean ± SEM, unless otherwise stated. Our LME model included in vitro capacitation (control, in vitro capacitation) as fixed effects and the pool of samples as the random part of the model.

## 3. Results

### 3.1. In Vitro Capacitation Increases the Live-Capacitated Sperm Ratio

**Figure 1:**
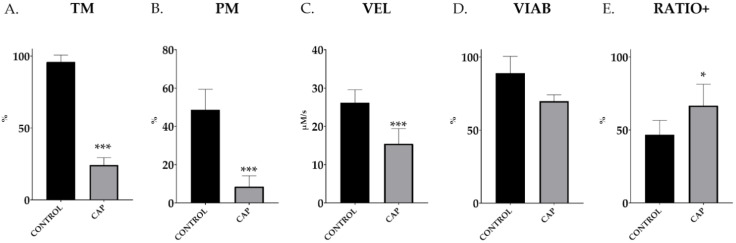
Total motility (TM), progressive motility (PM), velocity (VEL), membrane integrity (VIAB) and ratio of capacitated taking into account only the viable sperm (RATIO+) in control (CONTROL) and in vitro capacitated (CAP) boar ejaculated spermatozoa (*n* = 5). The results are shown as mean ± SEM. * depicts significant differences between CONTROL and CAP (* *p* < 0.05; *** *p* < 0.001).

In vitro exposure of commercial AI doses from a pool of ejaculated spermatozoa to elevated bicarbonate levels, under the assayed capacitation conditions (CAP; 38 °C, 5% CO_2_, 30 min), caused a significant reduction in total motility (96.0% ± 1.8 vs. 24.2% ± 1.9; *p* < 0.001; Figure 1A), fast progressiveness (48.7% ± 4.0 vs. 8.6% ± 2.1; *p* < 0.001; Figure 1B) and sperm velocity (26.1 ± 1.3 vs. 15.5 ± 1.5 µm/s; *p* < 0.001; Figure 1C) (CONTROL vs. CAP in all cases). In addition, the capacitating conditions for 30 min did not affect the membrane’s integrity, as depicted by YO-PRO-1^−^ spermatozoa (CONTROL, 89.0% ± 3.8 vs. CAP, 69.8% ± 1.5; *p* = 0.16; Figure 1D). The ratios of capacitated sperm within the live population (viable sperm, YO-PRO-1^−^ population) after in vitro capacitation were significantly higher than in the control group (*p* < 0.0083) (46.7% ± 3.3 vs. 66.7% ± 4.9; *p* = 0.0083; CONTROL vs. CAP; Figure 1E).

### 3.2. In Vitro Capacitation for 30 min Increases the Sperm Protein Kinase A Content

**Figure 2:**
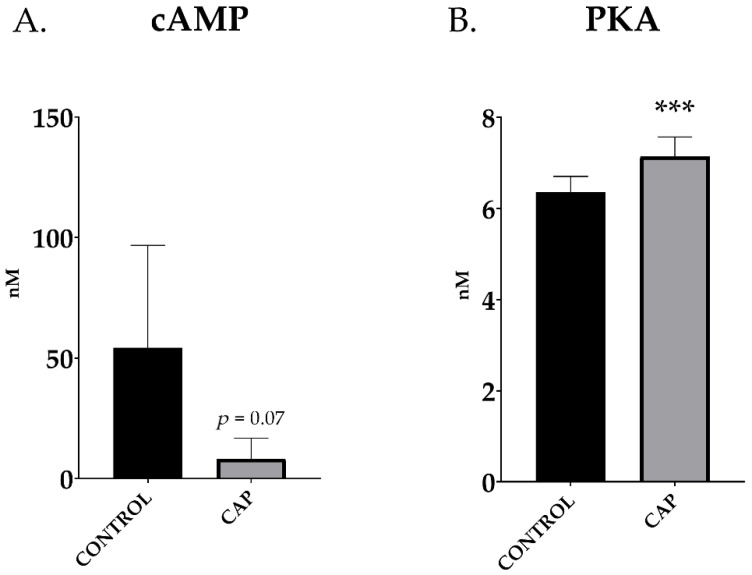
cAMP levels (**A**) and PKA levels (**B**) in control (CONTROL) and in vitro capacitated (CAP) boar ejaculated spermatozoa (*n* = 5). The results are shown as mean ± SEM. * depicts significant differences between CONTROL and CAP (*** *p* < 0.001).

Capacitated boar samples tend to lower its intracellular cAMP levels compared to control doses (54.2 ± 15.9 vs. 8.2 ± 3.2 nM; CONTROL vs. CAP; *p* > 0.05) although without significant differences (*p* = 0.07; Figure 2A). On the contrary, PKA increased in the sperm doses subjected to 30 min of capacitation; in this case, it increased significantly (6.36 ± 0.13 vs. 7.14 ± 0.16 nM/mL; CONTROL vs. CAP, *p* < 0.001; Figure 2B).

### 3.3. mRNA Expression of Catsper Subunits and TRPC Channels

**Figure 3:**
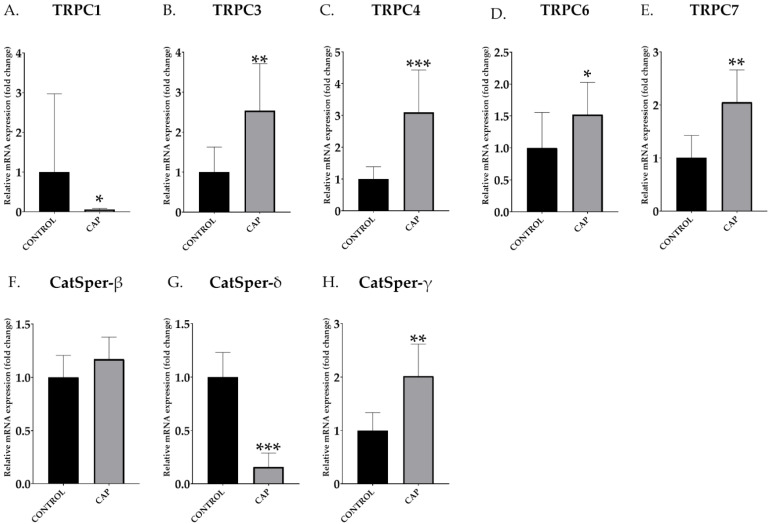
Relative abundance of mRNA transcripts of different TRPC and CatSper subunits control (CONTROL) and in vitro capacitated (CAP) boar ejaculated spermatozoa (*n* = 5). The values from mRNA expression have been represented relative to control, using a relative value of the control equal to 1. The results are shown as mean ± SEM. * Depicts significant differences between CONTROL and CAP (* *p* < 0.05, ** *p* < 0.01, *** *p* < 0.001).

TRPC (Figure 3A–E) and CatSper (Figure 3F–H) mRNA abundance data were obtained after qPCR. An increase in the relative abundance of mRNA transcript of TRPCs was observed in the in vitro capacitated boar spermatozoa (CAP) compared to the control samples (CONTROL) in most of the studied genes: *TRPC3* (CONTROL: 1.00 ± 0.57 vs. CAP: 2.54 ± 1.08; Figure 3B), *TRPC4* (CONTROL: 1.00 ± 0.35 vs. CAP: 3.09 ± 1.22; Figure 3C), *TRPC6* (CONTROL: 1.00 ± 0.51 vs. CAP: 1.52 ± 0.46; Figure 3D) and *TRPC7* (CONTROL: 1.00 ± 0.39 vs. CAP: 2.05 ± 0.55; Figure 3E); and *CatSper-γ* (CONTROL: 1.00 ± 0.27 vs. CAP: 2.43 ± 0.43; Figure 3H). *TRPC1* (CONTROL: 1.00 ± 0.80 vs. CAP: 0.06 ± 0.01; Figure 3A) and *Catsper-δ* (CONTROL: 1.00 ± 0.21 vs. CAP: 0.39 ± 0.16; Figure 3G) showed a significant decrease (*p* < 0.05) in the relative abundance of mRNA transcript in capacitated spermatozoa (*p* < 0.05). *CatSper-β* relative abundance increased in capacitated sperm compared to the control, although without significant differences (CONTROL: 1.00 ± 0.19 vs. CAP: 1.17 ± 0.19; Figure 3F).

## 4. Discussion

Because of bioethical limitations on human research, there is an urgent necessity for animal models to carry out comparative infertility studies [52]. In this regard, the porcine model includes crucial similarities with humans (including genetics, anatomy and physiology [53]) and is highly convenient for studies in reproductive physiology and health and for identifying diagnostic biomarkers [54]. In addition, between 5 and 10% of the breeding boars show fertility outcomes below the breed average and are considered sub-fertile [55], despite the rigorous reproductive controls to maximize the number of viable and motile spermatozoa in AI doses [56]. Therefore, there are continuous efforts to develop sophisticated semen analyses, aiding the elimination of subfertile sires [57]. Unfortunately, some crucial reproductive processes fall beyond the routine analysis performed in the porcine model, such as sperm capacitation.

The present study analyzed the effects of bicarbonate as a trigger for boar in vitro sperm capacitation on the abundance of mRNA for Ca^2+^ sperm channels and other sperm variables (motility, M540^+^/YO-PRO-1^−^ ratio and levels of PKA and cAMP) classically related to capacitation. Capacitation is a necessary process for mammalian sperm to fertilize the egg. A critical step is an increase in sperm cytoplasmic Ca^2+^ concentration during this process, which induces hyperactivated sperm motility [12,14,58]. Previous studies highlighted the presence of HCO_3_^−^ and BSA to elicit in vitro capacitation in pig sperm: e.g., 25 mM NaHCO3 and 0.4% BSA [59], and 36–38 mM NaHCO3 and 0.5% BSA [60,61,62,63,64]. Here, we use an in vitro capacitation medium previously established by our group [65].

We aimed to characterize the modification of the profile regarding sperm Ca^2+^ channels of boar spermatozoa during capacitation. To achieve this objective, relevant Ca^2+^ genes, including CatSper β, γ and δ genes and several TRPC genes (1, 3, 4, 6 and 7) mRNA levels were assessed in capacitated and non-capacitated boar spermatozoa in vitro by q-PCR. We successfully confirmed the modification of the relative abundance of mRNA transcripts of both CatSper subunits and TRPCs after in vitro incubation in controlled capacitation conditions. Regarding the analyses of *CatSper* β, δ and γ mRNA, we observed a reduction in the relative abundance of *CatSper*-δ. In contrast, *CatSper*-β and -γ relative abundances increased significantly in the case of *CatSper*-γ relative mRNA expression in capacitated sperm compared to the control. With respect to TRPC, we found a significant increase in the relative abundance of *TRPC3*, *TRPC4*, *TRPC6* and *TRPC7* mRNA transcripts after in vitro sperm capacitation. However, *TRPC1* relative mRNA expression decreased significantly after in vitro capacitation.

The results obtained from the analysis of both motility and velocity parameters decreased significantly after sperm in vitro capacitation but not viability. It has been reported that boar sperm capacitation is related to an increase in the intracellular ROS level [66] that it could reduce these parameters. Moreover, it has been studied those treatments containing high concentrations of bicarbonate (38 mM), with or without BSA, reduce the percentage of sperm with an intact membrane plasma and viability [67]. However, they conclude that high levels of bicarbonate reduce the time required for sperm to reach that capacitation status. Our results are in agreement with this and we checked that early sperm capacitation (30 min) reaches the capacitation state using high bicarbonate concentrations (37 mM NaHCO_3_), also in previous articles [65,68]. Moreover, merocyanine staining (M540^+^) confirmed the capacitation status of the spermatozoa as the number of alive spermatozoa positive for this dye (YO-PRO-1^+^) increased significantly after only 30 min of incubation. Moreover, our results from capacitation-like status induced by the in vitro capacitation show a significant increase in PKA while cAMP decreased. This reduction in cAMP agrees with the results of Battistone et al. 2013 [7] in human sperm samples with HCO_3_^−^ dependent capacitation. This study showed that cAMP level increased rapidly in the first minute of in vitro capacitation and then the values were progressively reduced. They did not detect significant differences between capacitated and non-capacitated samples, where [cAMP] is similar in both groups after 6 h [7]. Other authors have demonstrated that the response to bicarbonate is fast and cAMP levels increase within 60 s, followed by an increase in PKA activity and a rapid increase in this protein phosphorylation in human, mouse and boar sperm [69]. Our results agree with these observations, showing that [cAMP] counts were lower in capacitated sperm samples although without significant differences as early as 30 min under in vitro capacitation conditions in the presence of high concentrations of bicarbonate. Another fact relevant to our results is that the pig sperm proteome varies after capacitation when applying seminal plasma [70], and seminal plasma also modifies the endometrial transcriptome profile [71].

CatSper channels are essential for sperm to achieve its fertilization ability [65]. Failure in the hyperactivation of CatSper-deficient mice spermatozoa led to deficient egg’s vestment penetration [72]. In fact, CatSper-null spermatozoa were not able to fertilize ZP-intact oocytes, but only ZP-free oocytes, indicating a role of CatSper channels in the ability of spermatozoa to penetrate ZP [73]. Specifically, in CatSper-d-null mice spermatozoa, CatSper 1 was substantially reduced, and spermatozoa were infertile, suggesting that CatSper-d is essential for CatSper complex formation and ion channel function [30].

TRPC’s presence and distribution in spermatozoa vary across mammalian species [21]. Regarding TRPC channel’s relation to fertility in different species it is interesting to mention that in *C. elegans*, the oocytes fertilized by TRPC3-deficient spermatozoa showed a lack of local Ca^2+^ increase and a delay in the onset of the wave [74,75]. These results suggest that, along with its primary role in the fusion of spermatozoa and oocytes, TRPC3 induces Ca^2+^ waves in the fertilized oocytes. TRPC3 mutant spermatozoa are motile but do not fertilize the oocytes after gamete contact. Moreover, in *C. elegans*, TRPC3 is initially localized inside intracellular vesicles and then is translocated to the plasma membrane during sperm activation, coinciding with a marked increase in calcium influx [76]. TRPC1, 3, 4 and 6 are involved in the acrosomal reaction of human spermatozoa, but little is known about the specific mechanism of these channels in sperm capacitation processes that occur before fertilization. In addition, TRPC1, 4 and 6, which are mainly located in the flagellum, have been related to sperm mobility [35]. Interestingly, *TRPC1* transcript´s abundance decreased significantly after in vitro capacitation, which coincides with a motility decrease in our results, in agreement with previous studies on TRPC1, TRPC3 and TRPC6 distribution [35], although *TRPC3* and *TRPC6* increased.

The fact that mRNA increases during in vitro capacitation itself is an interesting result because the spermatozoon is transcriptionally silent [41,44,77], although some evidence suggested the contrary [78]. Not all mRNA comes from spermatogenesis, since some data support an epididymal origin of mRNA transmitted to maturing sperm by extracellular vesicles, and even a small proportion may also be synthesized by de novo transcription in mature sperm [79,80]. However, mRNA abundance in mature sperm does not necessarily correlate with the quantity of the protein expressed in the sperm, and more studies are needed to confirm the functional significance of the expression of Ca^2+^ channels´ mRNA in the spermatozoa. To the best of our knowledge, there is only one study describing changes in mRNA patterns in pigs after in vitro sperm capacitation [81]. Nevertheless, other authors proposed that the abundance of specific mRNAs could be used for estimating sperm quality in pig [82]. This correlates with studies in humans, where the use of microRNA expression has been related to different reproductive alterations [83,84]. Our results indicate that in vitro sperm capacitation elicits an increase in the relative abundance of mRNA transcript of almost all the studied Ca^2+^ channels. This finding confronts the prevalent idea of the spermatozoon being a transcriptionally silent cell, suggesting that spermatozoa not only provide paternal DNA at fertilization [77]. The mechanism underlying these changes in the mRNA pattern are beyond this study and still largely unknown, and they merit further research. In any case, we can rule out that these results arise from confounders such as variability in our reference gene *GAPDH* or interference of the sperm media in qPCR results. In the first case, the number of cycles for *GAPDH* was checked in the samples before and after capacitation, and we did not find significant differences. In the second case, it is unlikely that the composition of the capacitating medium could have affected the PCR, since we worked with sperm pellets after removing the medium by centrifugation. Therefore, our results are solidly based, irrespective of the underlying mechanism. Then, the changes in the abundance of mRNA transcripts after in vitro capacitation could be used as a new biomarker, being a useful tool to evaluate the overall quality status in commercial AI doses. Furthermore, more studies using boars of known fertility are needed to confirm if it could be used as a biomarker to discern between high-fertility and low-fertility males.

## 5. Conclusions

Our results indicate that in vitro sperm capacitation elicits an increase in the relative abundance of mRNA transcripts of almost all the studied Ca^2+^ channels, with the exception of *CatSper*-δ and *TRPC1*. More studies should be performed to depict the specific role of each channel in the boar and to elucidate the exact physiological significance of the changes on sperm mRNA cargo, as the spermatozoon has been considered as a transcriptionally silent cell.

## Data Availability

The data presented in this study are available in this article.
